# Complete Genome Sequence of Stenotrophomonas maltophilia Podophage Ponderosa

**DOI:** 10.1128/MRA.01032-19

**Published:** 2019-09-26

**Authors:** Alejandra Marquez, Heather Newkirk, Russell Moreland, Carlos F. Gonzalez, Mei Liu, Jolene Ramsey

**Affiliations:** a Center for Phage Technology, Texas A&M University, College Station, Texas, USA; DOE Joint Genome Institute

## Abstract

Stenotrophomonas maltophilia is a Gram-negative bacterium that is emerging as a multidrug-resistant global opportunistic pathogen. Here, we describe the genome of the T7-like S. maltophilia podophage Ponderosa, with 54 predicted protein-coding genes and a 493-bp terminal repeat.

## ANNOUNCEMENT

Stenotrophomonas maltophilia is a Gram-negative nonfermenting bacterium and an emerging multidrug-resistant pathogen (1). It can be found primarily in aqueous environments but also in soil and contaminated medical devices, such as catheters and breathing tubes. S. maltophilia is becoming increasingly resistant to antibiotics. As an alternative to antibiotics, phages are being examined for the treatment of S. maltophilia infections (2, 3), and here we describe a newly isolated phage, Ponderosa.

Bacteriophage Ponderosa was isolated from a filtered (0.2-μm pore size) water sample from the Shangani River in Kwekwe, Zimbabwe, based on its ability to grow on S. maltophilia (ATCC 17807). The host was cultured aerobically at 30°C in nutrient broth/agar (BD), and phages were isolated using the soft-agar overlay method described by Adams (4). Phage genomic DNA was purified as previously described in the shotgun library preparation modification to the Promega Wizard DNA clean-up system (5), and Illumina TruSeq Nano low-throughput libraries were made and sequenced on an Illumina MiSeq instrument with paired-end 250-bp reads using V2 500-cycle chemistry. Quality control on the 222,492 total sequence reads from the index containing the phage genome was done using FastQC (http://www.bioinformatics.babraham.ac.uk/projects/fastqc). Read trimming was performed using the FASTX-Toolkit v0.0.14 (http://hannonlab.cshl.edu/fastx_toolkit/). The phage genome was then assembled into a single contig at 29.3-fold coverage using SPAdes v3.5.0 (6). To ensure that the contig was closed, we performed Sanger sequencing on PCR products (forward primer, 5′-AGATGCGTTGTGGTTCTATCG-3′; reverse primer, 5′-GTCGTGCCATGTCATGTACT-3′) amplified off the genome ends. Protein-coding genes were predicted using GLIMMER v3.0 and MetaGeneAnnotator v1.0 (7, 8). ARAGORN v2.36 did not predict any tRNA genes in the Ponderosa genome (9). Gene function was then predicted using InterProScan v5.33-72, BLAST v2.2.31, TMHMM v2.0, and LipoP v1.0, with default settings, in the Galaxy and Web Apollo instances hosted at https://cpt.tamu.edu/galaxy-pub/ (10–15). BLAST searching was performed against the NCBI nonredundant, UniProtKB Swiss-Prot, and TrEMBL databases with a 0.001 maximum expectation value (16). Genome-wide DNA sequence similarity was calculated using progressiveMauve v2.4.0 (17). Rho-independent termination sites were annotated from TransTermHP v2.09 (18). The virion morphology was identified as a podophage by negative staining of the sample with 2% (wt/vol) uranyl acetate and the use of transmission electron microscopy at the Texas A&M Microscopy and Imaging Center (19).

The 42,612-bp double-stranded DNA genome has a coding density of 94.8%. Ponderosa has a G+C content of 59.98%, lower than the >66% G+C content of most *Stenotrophomonas* sp. strains (20). Analysis showed 54 predicted protein-coding genes, half of which were assigned functions. Ponderosa is most closely related to *Xylella* phage Paz (GenBank accession number KF626666), with 54.33% nucleotide identity and 37 shared proteins; both are T7-like in their genome size and organization. Ponderosa was predicted by PhageTerm (21) to have a 493-bp direct terminal repeat, and the genomes was re-opened according to synteny with that of phage T7 (GenBank accession number NC_001604).

The genomic organization in Ponderosa is also similar to *Pseudomonas* phage phiKMV (GenBank accession number NC_005045), another T7-like virus. The major structural genes annotated include four tail fibers (NCBI accession numbers QEG09761 to QEG09764).

### Data availability.

The genome sequence and associated data for phage Ponderosa have been deposited in GenBank under the accession number MK903280, BioProject accession number PRJNA222858, SRA accession number SRR8893604, and BioSample accession number SAMN11414489.
